# Complete mitochondrial genome sequence of *Aspergillus oryzae* RIB 127 and its comparative analysis with related species

**DOI:** 10.1080/23802359.2017.1375869

**Published:** 2017-09-09

**Authors:** Sajjad Asaf, Abdul Latif Khan, Muhammad Hamayun, Muhammad Aaqil Khan, Saqib Bilal, Sang-Mo Kang, In-Jung Lee

**Affiliations:** a School of Applied Biosciences, Kyungpook National University, Daegu, Republic of Korea;; b University of Nizwa, Nizwa, Oman;; c Department of Botany, Abdul Wali Khan University Mardan, Mardan, Pakistan

**Keywords:** Mitochondrial genome, *Aspergillus oryzae*, phylogeny, genome size

## Abstract

Here, we determined the complete sequence and annotation of the mitochondrial genome of *A. oryzae* (strain RIB 127). The complete mitochondrial genome is 29,202 base pairs (bp), with low GC content of 26.2%. Conserved genes identified include 26 transfer RNAs, the small and large ribosomal RNA subunits, and 14 protein-coding genes. Phylogenetic analysis based on the complete mitochondrial genome revealed that RIB 127 formed a single clade with two other *A. oryzae* species.

The *Aspergillus* genus comprises some of the most harmful, as well as, most beneficial fungal species. This genus includes various industrial producers of essential enzymes, antibiotics, and pharmaceuticals, which have had a substantial transformational influence on human health (Hoffmeister and Keller 2007). *Aspergillus oryzae* is a filamentous fungus, which has the capability of producing various amounts of hydrolytic enzymes (Zhao et al. 2013). Mitochondria are cellular organelles which play numerous, indispensable roles in all plant and animal cells (Basse 2010). The arrival of high-throughput sequencing technologies has assisted the rapid progress in the field of mitogenomics (Korab-Laskowska et al. 1998; O’brien et al. 2008).

In this study, we report and characterize the complete nucleotide sequence of strain RIB 127, obtained from American Type Culture Collection (ATCC) and stored in Crop physiology lab collection (accession number AO_SA93) at Kyungpook National University, South Korea. High-quality mitochondrial DNA was extracted using DNeasy Plant Mini Kit (Qiagen, Valencia, CA) and genome sequencing was performed using PacBio RS II sequencing platform. The complete mitochondrial genome sequence, with gene annotations, was submitted to GenBank with the accession number KY352472. To execute phylogenetic analysis, we included a total of 8 complete mt genomes from the genus *Aspergillus* in combination with the sequenced mt genome in this study. Phylogenetic analysis was performed using maximum likelihood (ML) and Bayesian inference (BI) analysis as implemented in MEGA6 and MrBayes 3.12, respectively (Ronquist and Huelsenbeck 2003; Tamura et al. 2013).

The mitochondrial genome of the RIB 127 strain has a simple genomic organization. It is a circular DNA molecule of 29,202 bp, with low GC content of 26.2%. Furthermore, the RIB 127 mt genome contains the typical set of mitochondrial RNA and protein genes characteristic of the majority of sequenced filamentous fungi mitochondrial genomes. The protein-coding genes include those for the electron transport complex I (*nad1*, *nad2*, *nad3*, *nad4*, *nad4*, *nad5,* and *nad6*), three subunits of complex IV, cytochrome oxidase (*cox1, cox2, and cox3*), ATP-synthase subunits (*atp6, atp8, and atp9*), and one subunit of complex III (*cob*). In addition to these 14 protein-coding genes, genes for small and large rRNA subunits and 26 tRNAs were also identified. In the phylogenetic tree, *A. oryzae* (RIB 127) formed a single clade with two other *A. oryzae* species (Figure 1). Use of mt genomes could be valuable for distinguishing between other closely related species or strains. This newly determined mitochondrial genome sequence of *A. oryzae* (RIB 127) provides valuable information that furthers our knowledge of genetic variation in the genus *Aspergillus*.

**Figure 1.**
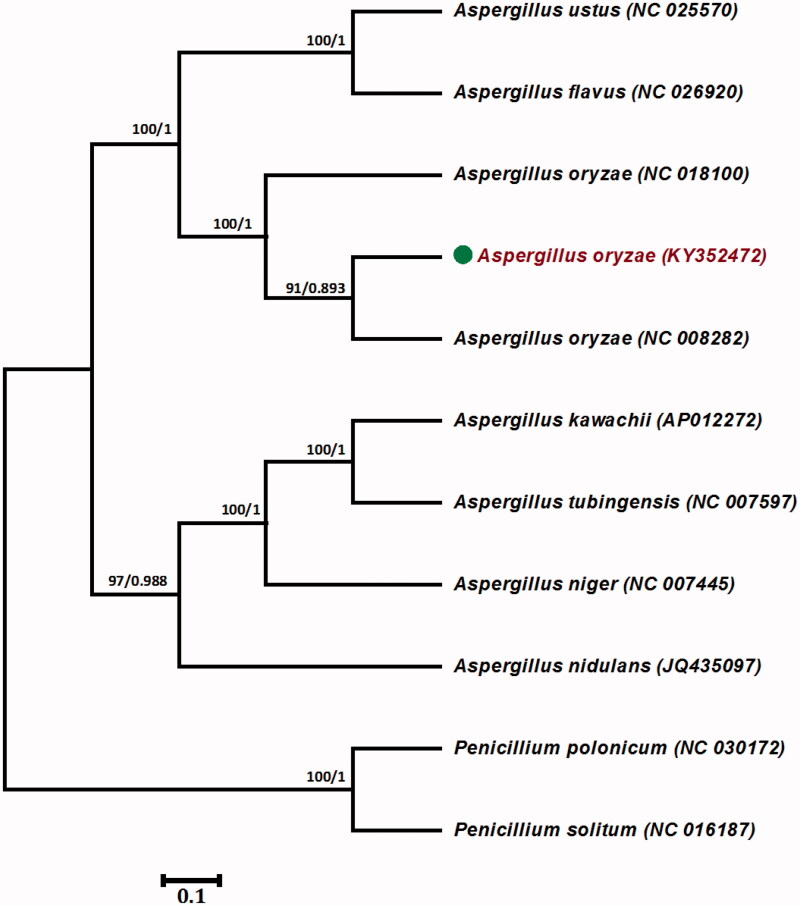
Phylogenetic tree based on complete mitochondrial genomes of nine species from the genus *Aspergillus* constructed using two different methods: maximum likelihood (ML) and Bayesian inference (BI). Numbers above the branches are the bootstrap values of ML and posterior probabilities of ML and BI, respectively. Dot represents the position of *Aspergillus oryzae* (KY352472).
